# When ultrasound-guided catheterization is useless: back to landmarks!

**DOI:** 10.1186/cc13986

**Published:** 2014-07-11

**Authors:** Raphaël Giraud, Karim Bendjelid

**Affiliations:** 1 Intensive Care Service, Geneva University Hospitals, 4 Rue Gabrielle Perret-Gentil, Geneva 14, CH-1211, Switzerland; 2 Faculty of Medicine, University of Geneva, Rue Michel Servet 1, Geneva, 1205, Switzerland; 3 Geneva Hemodynamic Research Group, University of Geneva, Rue Michel Servet 1, Geneva, 1205, Switzerland

## 

A study by Maizel and colleagues [1] in a recent issue of *Critical Care* shows that a resident skilled at inserting a central venous catheter (CVC) via the ultrasound-guided (UG) technique may face difficulties inserting a CVC via the anatomical landmark (LM) technique. Because several studies have demonstrated significantly increased safety, effectiveness, and efficiency of UG vascular access, as compared with cannulation by anatomical LMs, the UG technique became the more broadly recommended procedure [2-4]. However, as residents are trained only in this technique, they are no longer able to perform the LM technique, even when ultrasound is not available or applicable.

In fact, we recently had a patient with extensive subcutaneous emphysema requiring veno-venous extracorporeal membrane oxygenation. Percutaneous cannula implantation, usually performed under ultrasound guidance, has been inserted using the LM technique. Because of emphysema, no visualization of veins was possible (Figure 1).

**Figure 1:**
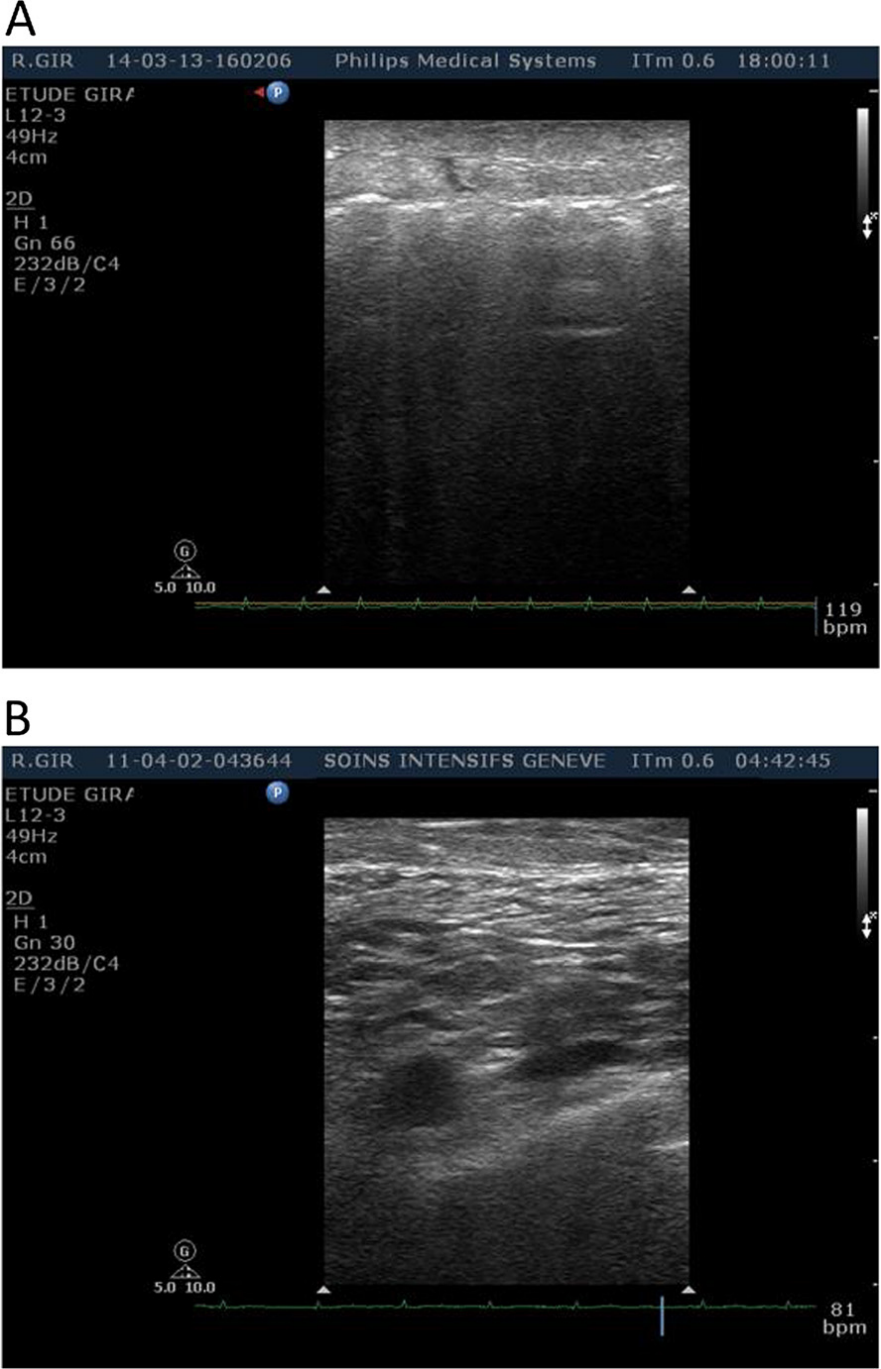
**Ultrasound images of the neck for the internal jugular vein location during vascular access puncture (A) with and (B) without subcutaneous emphysema.** An impossible ultrasound guidance (A) and a possible ultrasound guidance (B) are shown.

Despite international recommendations, it seems to be essential to avoid the UG one-way dogmatic approach and continue to train our young colleagues to insert the CVC via a conventional technique because in certain situations in which ultrasound guidance cannot be used, cannulation remains vital for the patient. Thus, if the clinical situation is favorable (anatomy and coagulation are normal, and the patient is not overweight), young doctors could make a puncture test according to the LM technique. The UG technique would be automatically performed in case of failure of the first and single puncture. In this way, the LM technique will continue to be taught so that all doctors can insert catheters in situations when ultrasound is not usable.

## Abbreviations

CVC: Central venous catheter; LM: Landmark; UG: Ultrasound-guided.

## Competing interests

The authors declare that they have no competing interests.
